# Case Report: Adjunctive hemoadsorption therapy in a patient with Takotsubo cardiomyopathy and hyperinflammation

**DOI:** 10.3389/fmed.2025.1680632

**Published:** 2025-09-23

**Authors:** Jose O. Castro

**Affiliations:** Department of Critical Care Medicine, Pacifica Salud Hospital, Panama City, Panama

**Keywords:** extracorporeal blood purification, hemoadsorption technology, CytoSorb, Takotsubo cardiomyopathy, multiorgan dysfunction

## Abstract

**Introduction:**

Takotsubo cardiomyopathy (TCM) is a transient, stress-related cardiac dysfunction that mimics acute coronary syndrome and is typically seen in postmenopausal women. While emotional and physical stressors are common triggers, inflammatory or infectious stimuli have also been proposed, although the underlying mechanisms remain incompletely understood. In the context of critical illness, systemic inflammation may exacerbate cardiac dysfunction and contribute to multiorgan failure. Hemoadsorption has emerged as a promising adjunctive therapy option aimed at mitigating hyperinflammation by removing circulating cytokines and other inflammatory mediators, and has shown potential benefit in various inflammatory conditions.

**Case description:**

A 77-year-old woman with hypertension, diabetes, and a permanent pacemaker presented with acute dyspnea and subsequently experienced cardiac arrest. Post-resuscitation, she was found to have anterolateral ST elevations, apicomedial akinesis, and severely reduced LVEF (22%), consistent with Takotsubo cardiomyopathy. Despite appropriate antimicrobial therapy and supportive care, she developed worsening fever, marked hemodynamic instability, hyperlactatemia (14 mmol/L), atrial fibrillation, raising suspicion for a systemic hyperinflammatory response. Hemoadsorption therapy with CytoSorb was initiated in a stand-alone configuration, alongside corticosteroids and targeted temperature management. The patient showed marked improvement in hemodynamics, organ function, and urine output within 48 h, without the need for renal replacement therapy. She was later extubated and discharged home.

**Conclusion:**

This case highlights a rare presentation of suspected inflammation-triggered Takotsubo cardiomyopathy complicated by rapid clinical deterioration. The use of CytoSorb hemoadsorption was associated with stabilization of vital parameters and recovery of organ function. Although causality cannot be confirmed, this case supports the early consideration of hemoadsorption as a feasible and potentially beneficial adjunct in select critically ill patients with suspected hyperinflammation. Further research is needed to clarify its role in TCM and related syndromes.

## Introduction

Takotsubo cardiomyopathy (TCM), also known as stress-induced or “broken heart” syndrome, is an acute and typically reversible form of heart failure characterized by transient left ventricular systolic dysfunction in the absence of significant coronary artery disease. It accounts for approximately 1–2% of all patients presenting with suspected acute coronary syndrome and predominantly affects postmenopausal women (1, 2). While emotional and physical stressors are well-known precipitants, infectious triggers have also been increasingly recognized, particularly in critically ill patients (3).

In some cases, TCM may present in conjunction with or be triggered by a systemic hyperinflammatory state, such as cytokine release syndrome (CRS). CRS is a life-threatening condition marked by excessive activation of the immune system, resulting in a cascade of pro-inflammatory cytokine release. It has been described in a variety of clinical contexts, including viral infections (e.g., COVID-19), sepsis, autoimmune disorders, and cellular immunotherapies such as CAR-T cell therapy (4). Severe CRS can lead to rapid multiorgan failure, hemodynamic collapse, and death if not promptly recognized and managed. The pathophysiology involves a self-amplifying loop of immune activation, endothelial dysfunction, and tissue damage (5).

The diagnosis of CRS is clinical, supported by biomarker elevation (e.g., interleukin-6, ferritin, C-reactive protein), and requires exclusion of alternative causes such as persistent infection. Management strategies typically include hemodynamic stabilization, organ support, and in some cases, immunomodulatory therapies such as corticosteroids, IL-6 inhibitors (e.g., tocilizumab), or extracorporeal therapies aimed at cytokine removal (6).

Extracorporeal blood purification techniques, including hemoadsorption, have gained attention as adjunctive therapies in the management of hyperinflammatory syndromes. These therapies aim to modulate the immune response by reducing circulating levels of inflammatory mediators. Among these, CytoSorb (CytoSorbents Corporation, United States) is the most widely used device, approved in the European Union for the removal of cytokines and other inflammatory mediators from whole blood. However, the optimal timing and patient selection for this intervention remain under investigation. This case report describes a critically ill patient with suspected infection-triggered Takotsubo cardiomyopathy and hyperinflammation, who showed significant clinical improvement following the early initiation of stand-alone CytoSorb hemoadsorption therapy.

## Case description

A 77-year-old female patient with a medical history of arterial hypertension, type 2 diabetes mellitus, and a permanent pacemaker was brought to the emergency department by family members due to acute onset of dyspnea. Upon arrival, she presented with impaired consciousness, respiratory distress, and hypoxemia. Shortly thereafter, she experienced cardiac arrest with a non-shockable rhythm. Cardiopulmonary resuscitation was initiated, and return of spontaneous circulation (ROSC) was achieved after 2 min.

**Figure 1:**
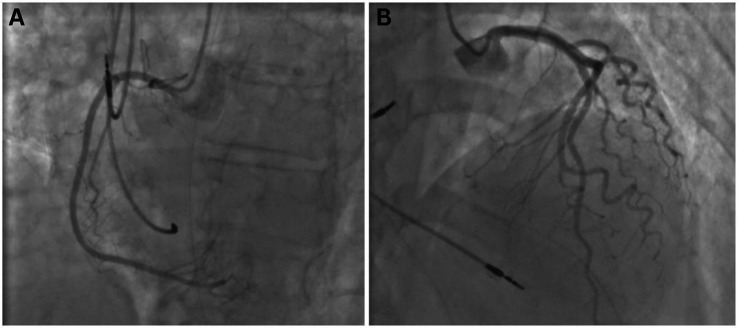
Coronary angiography performed following cardiopulmonary resuscitation at hospital admission, demonstrating no significant coronary artery stenosis or obstructive lesions in the right **(A)** or left **(B)** coronary systems. Ventriculography was not obtained.

Immediately post-resuscitation, electrocardiogram revealed ST-segment elevation in the anterolateral wall. Coronary angiography was performed and confirmed the absence of significant coronary artery stenosis or obstructive lesions (Figure 1). A computed tomography pulmonary angiogram showed no evidence of pulmonary embolism but revealed diffuse bilateral alveolar infiltrates, predominantly affecting the right lower lobe, suggestive of pulmonary edema or pneumonia. Transthoracic echocardiography showed a left ventricular ejection fraction (LVEF) of 22% with akinesia of apical and mid-ventricular segments and no evidence of left ventricular hypertrophy, raising suspicion for Takotsubo cardiomyopathy.

Microbiological analysis identified methicillin-sensitive *Staphylococcus aureus* by PCR from endotracheal secretions. There was no evidence of renal or hepatic dysfunction at admission, and nasal swab testing for respiratory viruses was negative. The patient was diagnosed with cardiogenic shock in the context of a likely pulmonary infection as the triggering factor.

She was admitted directly to the intensive care unit, intubated, mechanically ventilated, and started on furosemide boluses every 8 h, along with continuous low-dose dobutamine and norepinephrine infusion. Empiric antibiotic therapy with cefepime and vancomycin was initiated and later de-escalated to cefazolin following final culture results and in consideration of a documented penicillin allergy.

Within 48 h, the patient showed signs of neurological recovery with preserved responsiveness and ability to follow commands. There was radiographic improvement in pulmonary infiltrates, attributed to achievement of a negative fluid balance, and a reduced requirement for norepinephrine. However, the patient developed persistent, progressively worsening fever despite appropriate antibiotic therapy. Further investigation for infectious sources, including a whole-body CT scan, yielded no significant findings. Transesophageal echocardiography excluded pacemaker-related endocarditis, and repeated blood cultures remained negative. Discontinuation of potentially pyrogenic medications did not result in clinical improvement.

**Figure 2:**
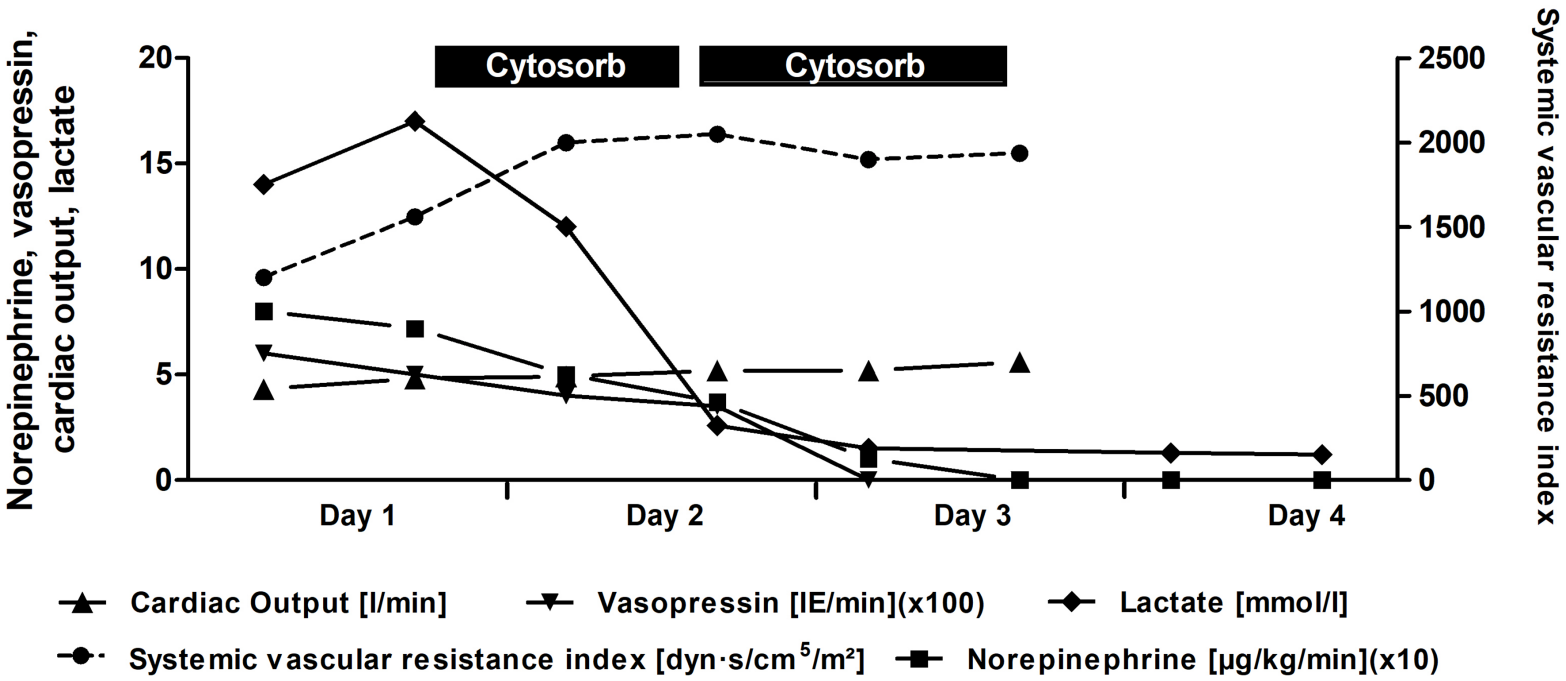
Course of vasopressor requirements, hemodynamic parameters, and lactate levels during and after CytoSorb hemoadsorption therapy. For improved visualization of trends, norepinephrine and vasopressin doses are displayed multiplied by factors of 10 and 100, respectively.

**Figure 3:**
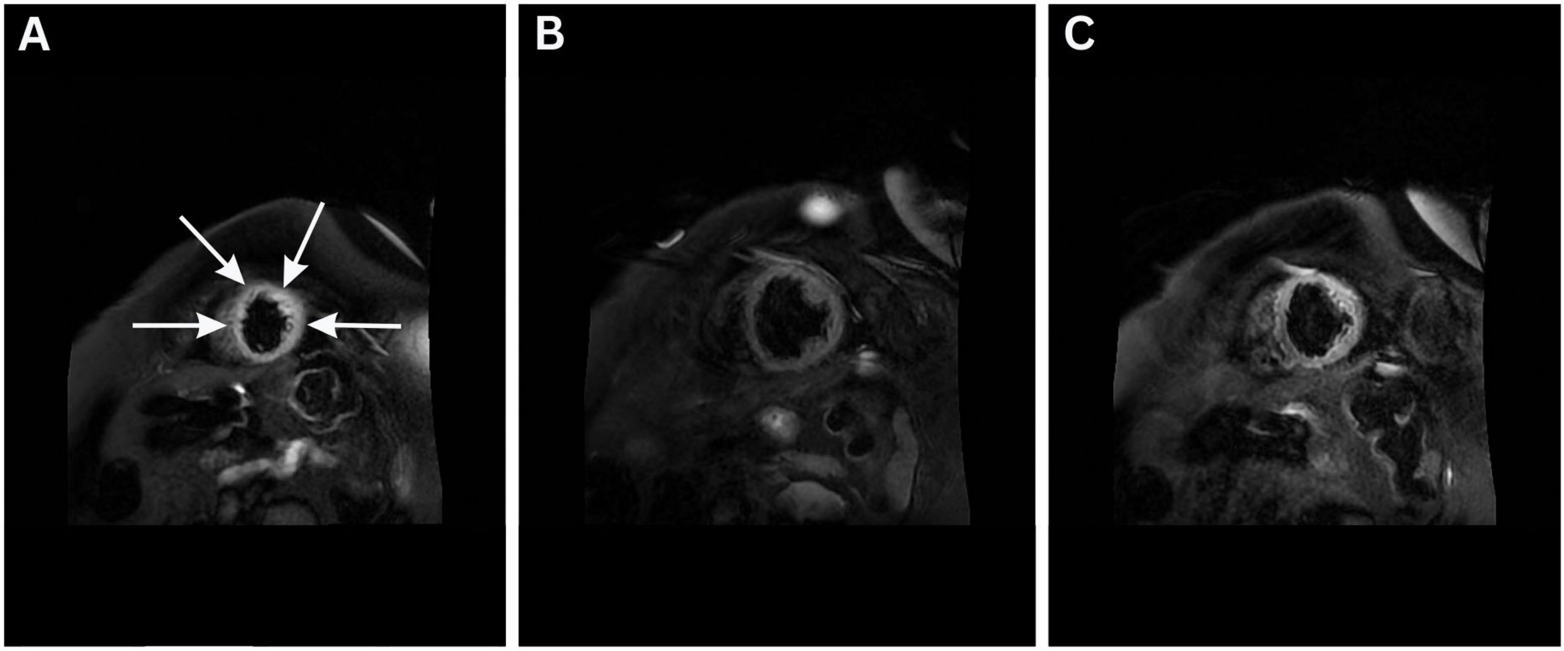
Cardiac magnetic resonance imaging performed on the 6th day of admission **(A–C)**, T2 STIR images obtained showing apical and mid-ventricular edema (arrows in A) and akinesia with severely reduced left ventricular ejection fraction (LVEF-3D: 22%). Volumetric analysis revealed normal left ventricular size (LVEDV/BSA = 80 mL/m^2^) and no evidence of left ventricular hypertrophy. These findings are consistent with Takotsubo cardiomyopathy and help exclude infarction and myocarditis as differential diagnoses.

On the sixth day of admission, the patient developed a high-grade fever (up to 40.5 °C), new-onset rapid atrial fibrillation (135–140 bpm), increased respiratory drive with ventilator dyssynchrony, and increasing vasopressor requirements, necessitating the addition of vasopressin (Figure 2). Cardiac function remained unchanged on echocardiography. Cardiac magnetic resonance imaging provided further support for the diagnosis of Takotsubo cardiomyopathy (Figure 3). The left ventricle was of normal size on volumetric analysis (LVEDV/BSA = 80 mL/m^2^), with akinesia of the apical and medial segments and severely reduced systolic function (LVEF-3D: 22%). There was no evidence of left ventricular hypertrophy. The left atrium appeared dilated, while the right ventricle demonstrated preserved function (TAPSE = 20 mm) and the left atrium was also reported as normal in size. Importantly, late gadolinium enhancement (LGE) revealed no signs of intramyocardial fibrosis, inflammatory changes, infiltrative disease, or scarring. No pleural or pericardial effusions were detected. All major intrathoracic vascular structures including the main pulmonary artery, pulmonary branches, ascending and descending aorta, superior and inferior vena cava, and coronary sinus were of normal diameter. Concurrent laboratory tests revealed severe metabolic acidosis, elevated lactate (up to 14 mmol/L), hyperglycemia, and markedly elevated inflammatory markers including ferritin (1,253 ng/mL; reference range: 11–200 ng/mL), urine output declined to 5 mL/h with creatinine levels still being normal (0.91 mg/dL). Central venous oxygen saturation was 75%, and the patient exhibited profound hemodynamic instability with significantly decreased systemic vascular resistance (SVRI) of 1,200 dynes·s/cm^5^ despite vasopressor support.

Given this acute clinical picture, targeted temperature management was initiated via intravascular cooling, in combination with intravenous pulse-dose corticosteroids. In parallel, hemoadsorption therapy using CytoSorb was started in stand-alone configuration (blood flow rate 200 mL/min with anticoagulation using heparin at 600 units/h). Two consecutive CytoSorb treatments were administered over a total of 44 h (1st treatment 18 h, 2 h pause interval, 2nd treatment 24 h).

Following the initiation of this multimodal intervention, the patient exhibited a marked decrease in vasopressor requirements paralleled by a stabilization in hemodynamic parameters and a normalization in lactate levels (Figure 2). After discontinuation of CytoSorb treatment, the patient remained hemodynamically stable. Urine output returned to normal with 50 mL/h at the end of CytoSorb therapy and 100 mL/h one day later. This was parallelled by a temporary slight increase of creatinine values (peak levels of 1.95 mg/dL) which normalized over time (0.81 mg/dL three days after end of CytoSorb therapy) without the need for renal replacement therapy. Also ferritin levels had meanwhile halved by then (660 ng/mL). Subsequently, however, the patient developed ventilator-associated pneumonia, which was effectively treated by conservative therapy. She was successfully extubated on the 13th day of mechanical ventilation. A minor gastrointestinal bleeding episode occurred and was managed conservatively with proton pump inhibitors administered every 12 h.

A follow-up transthoracic echocardiogram was performed 23 days after ICU admission, revealing normalization of left ventricular diameter and volumes, with persistent akinesia of the apical segments, hypokinesia of the mid-ventricular segments, and hypercontractility of the basal segments, consistent with a stress cardiomyopathy motion pattern. Left ventricular ejection fraction had increased to 38–41%, indicating a clear improvement compared to the initial echocardiogram. Additional findings included mild concentric hypertrophy (107 g/m^2^), type 2 diastolic dysfunction with normal filling pressures, and mild aortic and mitral insufficiency with structurally normal valves. The left atrium was slightly dilated (38 mL/m^2^), while the right atrium remained within normal limits. Mild tricuspid regurgitation was observed, with normal valve morphology and an estimated pulmonary artery systolic pressure of 35 mmHg. All other echocardiographic parameters were unremarkable. The patient was discharged home on the 24th day of hospitalization with arrangements for private nursing care and ongoing rehabilitation due to critical illness-associated myopathy.

## Discussion

This case illustrates a rare clinical presentation of Takotsubo cardiomyopathy associated with a suspected cytokine release syndrome, leading to profound hemodynamic instability and multiorgan dysfunction. The initiation of CytoSorb therapy, alongside targeted temperature management and corticosteroids, was associated with a clear improvement in the patient’s clinical trajectory. The observed stabilization of metabolic, inflammatory, and organ function parameters suggests a potential role for extracorporeal cytokine adsorption in similar critical care scenarios.

Although Takotsubo cardiomyopathy often follows emotional or physical stress, infectious triggers have also been reported, particularly in the context of systemic inflammation or sepsis (1). In this case, a pulmonary infection with *Staphylococcus aureus* appears to have precipitated cardiogenic shock and Takotsubo-like wall motion abnormalities, confirmed by echocardiography and supported by the absence of coronary lesions. The diagnosis of Takotsubo cardiomyopathy in this case was supported by multiple imaging modalities. Coronary angiography confirmed the absence of obstructive coronary artery disease, and cardiac magnetic resonance imaging revealed apical and mid-ventricular akinesia with severely reduced LVEF (22%), but no evidence of myocardial fibrosis, inflammation, or scarring on late gadolinium enhancement. These findings helped to exclude myocardial infarction or myocarditis as differential diagnoses. Furthermore, a follow-up echocardiogram demonstrated partial recovery of left ventricular function (LVEF 38–41%) and improvement in regional wall motion abnormalities, consistent with the expected course of stress cardiomyopathy. Together, these findings fulfilled key diagnostic criteria for Takotsubo cardiomyopathy, including transient ventricular dysfunction in the absence of coronary obstruction or irreversible myocardial damage.

The patient’s subsequent deterioration, marked by high fever, metabolic acidosis, hyperlactatemia, rising inflammatory markers (ferritin), and hemodynamic collapse, was consistent with a cytokine release syndrome (CRS). CRS has been increasingly recognized not only in oncologic or viral contexts (e.g., CAR-T cell therapy, severe COVID-19) but also in bacterial infections and critical illness, where dysregulated immune responses drive organ dysfunction (4, 5, 7).

While inotropes are generally used with caution in Takotsubo cardiomyopathy due to concerns about catecholamine-induced myocardial stress and potential for dynamic left ventricular outflow tract obstruction (LVOTO), our patient’s echocardiographic evaluations did not reveal LVOTO. Furthermore, the clinical picture was dominated by distributive (vasoplegic) shock rather than isolated cardiogenic shock, as evidenced by low systemic vascular resistance, elevated inflammatory markers, and relatively preserved right ventricular function. This justified the cautious administration of low-dose dobutamine alongside vasopressors for hemodynamic support under close monitoring. Mechanical circulatory support (MCS) options such as venoarterial ECMO or Impella are available at our center; however, in this case, the patient experienced sudden hemodynamic collapse at the bedside, making the initiation of MCS logistically and temporally unfeasible. Thus, the clinical decision-making prioritized rapid stabilization using pharmacologic agents combined with early extracorporeal cytokine removal.

CytoSorb is a hemoadsorption device designed to remove circulating cytokines and inflammatory mediators. Though evidence is still evolving, its use in sepsis, cardiac surgery, and cytokine storm syndromes has shown promise in improving hemodynamic stability and reducing vasopressor dependency (8, 9). In this case, CytoSorb therapy was initiated in a stand-alone configuration during a period of escalating inflammatory and hemodynamic stress. It was associated with rapid improvement in metabolic parameters, urine output, and a marked reduction in vasopressor requirements—outcomes that align with previous reports on CytoSorb use in hyperinflammatory states (10). In this case, CytoSorb therapy was initiated in a stand-alone configuration, outside of dialysis or ECMO circuits, using a dedicated extracorporeal blood pump setup. Importantly, this intervention was applied before overt renal failure occurred. Renal function was preserved throughout the ICU stay, and urine output normalized early in the course of therapy. This case underscores a critical consideration in managing CRS: waiting for irreversible organ dysfunction, such as established acute kidney injury requiring renal replacement therapy (RRT), may limit the window for effective immunomodulation. Early application of adjunctive therapies like CytoSorb, based on clinical trajectory, biomarker elevation, and physiologic deterioration, may help interrupt the vicious cycle of cytokine-driven injury, potentially altering the course of disease.

CytoSorb was found to be safe, easy to implement within existing ICU workflows, and well-tolerated. While the temporal association suggests benefit, definitive conclusions about causality cannot be drawn from a single case. Nonetheless, the clinical course is indicative of a potential therapeutic role for extracorporeal cytokine adsorption in patients with suspected cytokine storm and refractory shock.

While causal relationships cannot be definitively established, this case highlights the potential role of extracorporeal cytokine removal in altering the trajectory of fulminant hyperinflammation before irreversible organ damage occurs.

## Data Availability

The original contributions presented in the study are included in the article/supplementary material, further inquiries can be directed to the corresponding author.
